# Dynamic Changes in the Aorta During the Cardiac Cycle Analyzed by ECG-Gated Computed Tomography

**DOI:** 10.3389/fcvm.2022.793722

**Published:** 2022-05-19

**Authors:** Wenying Zhu, Yingliang Wang, Yang Chen, Jiacheng Liu, Chen Zhou, Qin Shi, Songjiang Huang, Chongtu Yang, Tongqiang Li, Bin Xiong

**Affiliations:** ^1^Department of Radiology, Union Hospital, Tongji Medical College, Huazhong University of Science and Technology, Wuhan, China; ^2^Hubei Key Laboratory of Molecular Imaging, Wuhan, China

**Keywords:** aorta, diameter, dynamic changes, cardiac cycle, oversizing

## Abstract

**Background:**

To characterize the difference in aortic dimensions during the cardiac cycle with electrocardiogram (ECG)-gated computed tomography angiography (CTA) and to determine whether other parameters in comparison to diameter could potentially provide a more accurate size reference for stent selection at the aortic arch and the proximal thoracic descending aorta.

**Methods:**

The CTA imaging of 90 patients during the cardiac cycle was reviewed. Three anatomic locations were selected for analysis (level A: 1 cm proximal to the innominate artery; level B: 1 cm distal to the left common carotid artery; and level C: 1 cm distal to the left subclavian artery). We measured the maximum diameter, the minimum diameter, the lumen area, the lumen perimeter, and the diameter derived from the lumen area, and the changes of each parameter at each level during the cardiac cycle were compared.

**Results:**

The mean age was 60.9 ± 12.4 years (range, 16–78 years). There was a significant difference in the aortic dimensions during the cardiac cycle (*p* < 0.001). The diameter derived from the lumen area at all three levels was changed least over time when compared to the area, perimeter, and the maximum aortic diameter (all *p* < 0.01).

**Conclusion:**

The aortic dimensional differences during the cardiac cycle are significant. The aortic diameter derived from the lumen area over other parameters may provide a better evaluation for selecting the size of the stent at the aortic arch and the proximal thoracic descending aorta. A prospective study comparing these different measurement parameters regarding the outcomes is still needed to evaluate the clinical implications.

## Introduction

Aortic dissection is a catastrophic event associated with a variety of clinical manifestations. Thoracic endovascular aortic repair (TEVAR) has been extensively used to treat aortic dissection since it was firstly reported in 1999 (1). Compared to open surgery, TEVAR has less morbidity, decreased hospital stay, and improved outcomes (2–4). However, TEVAR still poses stent-related complications resulting from inadequate oversizing. Choosing too small a stent dimension can cause endoleak and device migration, whereas sizing too much may lead to infolding of the stent or local injury of the aortic wall (5, 6). Thus, appropriate stent size is crucial to avoid these adverse events and receive optimal long-term outcomes.

At present, the maximum aortic diameter in the proximal landing zone based on non-gated computed tomography angiography (CTA) is usually used as a reference for stent selection. There indeed exist some problems with this method. Firstly, static CTA imaging was obtained at random phases during the cardiac cycle, which does not take the dynamic changes of dimension into consideration and hence can result in an inappropriate measurement for stent reference. Some studies have reported statistically significant changes in the aortic dimension between the systolic and diastolic phases though some of the study results are controversial (7–9). Secondly, whether the maximum aortic diameter is the optimal reference for stent selection is still controversial. Belvroy et al. (10) reported that changes in ascending aortic diameter during the cardiac cycle are larger than in the area and it may be more accurate to measure the area when selecting the size of the stent. Meanwhile, Parodi et al. (11) found that the selection of the aortic diameter based on the lumen area over direct measurements of the diameter could provide a better evaluation of the optimal oversizing of the stent in the descending aorta.

Accurate measurement of aortic dimension is of significant importance for selecting the appropriate size of the stent-graft, and size mismatch between the aorta and stent could lead to stent migration, infolding, kinking, endoleaks and aortic wall injury, and subsequent poor prognosis (12, 13). However, the aortic dimension changes during the cardiac cycle due to cardiac pulsatility and aortic wall compliance. Thus, the electrocardiogram (ECG)-gated CTA that could obtain images at different time points during the cardiac cycle was used to study the changes in aortic dimensions (14).

The number of studies using ECG-gated CTA to investigate the dynamic changes in aortic dimensions during the cardiac cycle was limited, the reported aortic segmentations mostly focused on the ascending aorta or descending thoracic aorta, and the selected parameters were varied in current literature. Thus, the purpose of this study was to characterize the difference in the aortic dimensions between systolic and diastolic phases with ECG-gated CTA and to determine whether other parameters, such as area or perimeter in comparison to diameter, could potentially provide a more accurate size reference for stent selection at the aortic arch and the proximal thoracic descending aorta. In the present study, the three measurement levels we choose were the most common proximal landing zones at the aortic arch and proximal descending thoracic aorta, which made them more applicable in clinical practice. In addition, our study included all parameters in current literature and thus our comparison was more comprehensive.

## Materials and Methods

### Study Population

**Figure 1:**
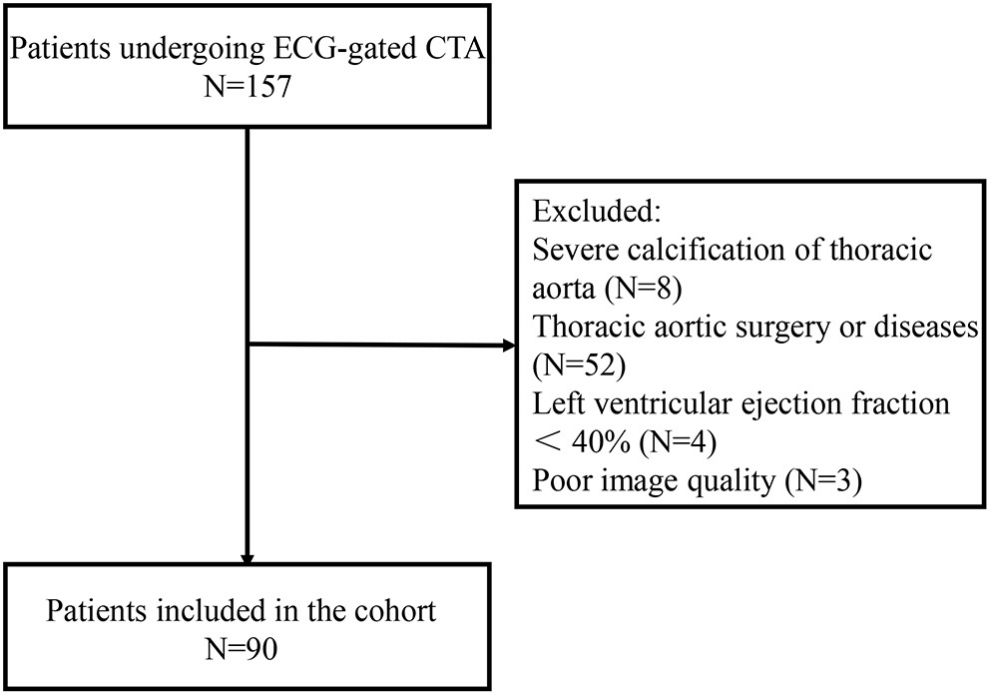
Flowchart of inclusion of patients.

A total of 90 consecutive patients between December 2018 and July 2019 were retrospectively analyzed in this study. The patients underwent ECG-gated CTA with a retrospective gating due to valvular diseases, coronary arterial diseases, and abdominal aortic aneurysm or dissection. Patients with severe calcification of thoracic aorta, thoracic aortic disease, previous thoracic aortic surgery, and left ventricular ejection fraction <40% were excluded. CTA with cardiac or respiratory artifacts preventing clinical assessment of aortic dimensions was also excluded (Figure 1).

### Morphometric Parameter Measurements

All CTA scans were performed using a Siemens Somatom Force Dual Source CT Scanner (Siemens Healthcare GmbH, Forchheim, Germany) with ECG gating. Images were acquired during a single breath-hold phase (<20 s) and the entire thoracic aorta was imaged. The imaging protocol was set at 0.6 mm collimation, 0.5-s rotation time, and a pitch of 3.2. Radiation exposure parameters were 100 kVp with different effective milliampere-second values (set automatically by the software), resulting in a CT dose index (CTDI_vol_) of 3.48 ± 1.16 mGy, dose length product (DLP) of 297 ± 43.8 mGy^.^cm, and effective dose (ED) of 3.68 ± 0.95 mSv. Intra-vascular non-ionic contrast (0.8 ml/kg) (Iodixanol; Hengrui, China) was intravenously injected with a power injector at 4 ml/s. The images were reconstructed with a slice thickness of 0.75- and 0.5-mm intervals. All images were then transferred to a separate workstation that was equipped with CT post-processing software (Syngo.via, Siemens Healthcare GmbH, Erlangen, Germany). The syngo.CT Vascular Analysis (Syngo.via, Siemens Healthcare GmbH, Erlangen, Germany) is a commercially available software. A protocol for the measurement of different parameters using the centerline method was established (15). This protocol was set to ensure measurements during the systolic and diastolic phases. These phases were defined by assessing the lowest and highest left ventricular volumes, respectively, and were used to make confident measurements during the cardiac cycle. The R-R interval between 30–40% and 70–80% corresponded to systolic and diastolic phases for all the patients, respectively.

**Figure 2:**
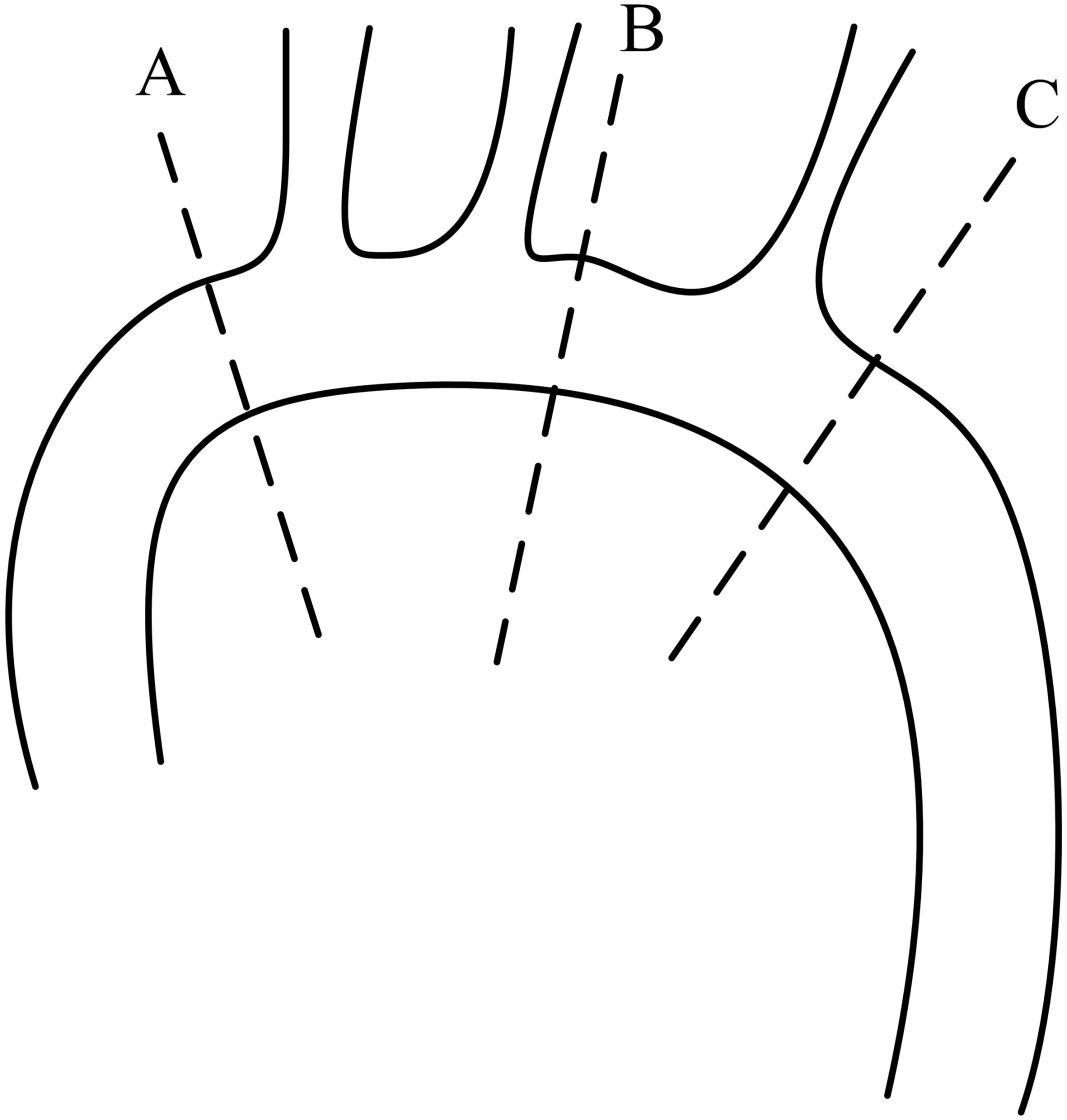
Locations of the measurements in the aorta; level A: 1 cm proximal to the innominate artery; level B: 1 cm distal to the left common carotid artery; and level C: 1 cm distal to the left subclavian artery.

**Figure 3:**
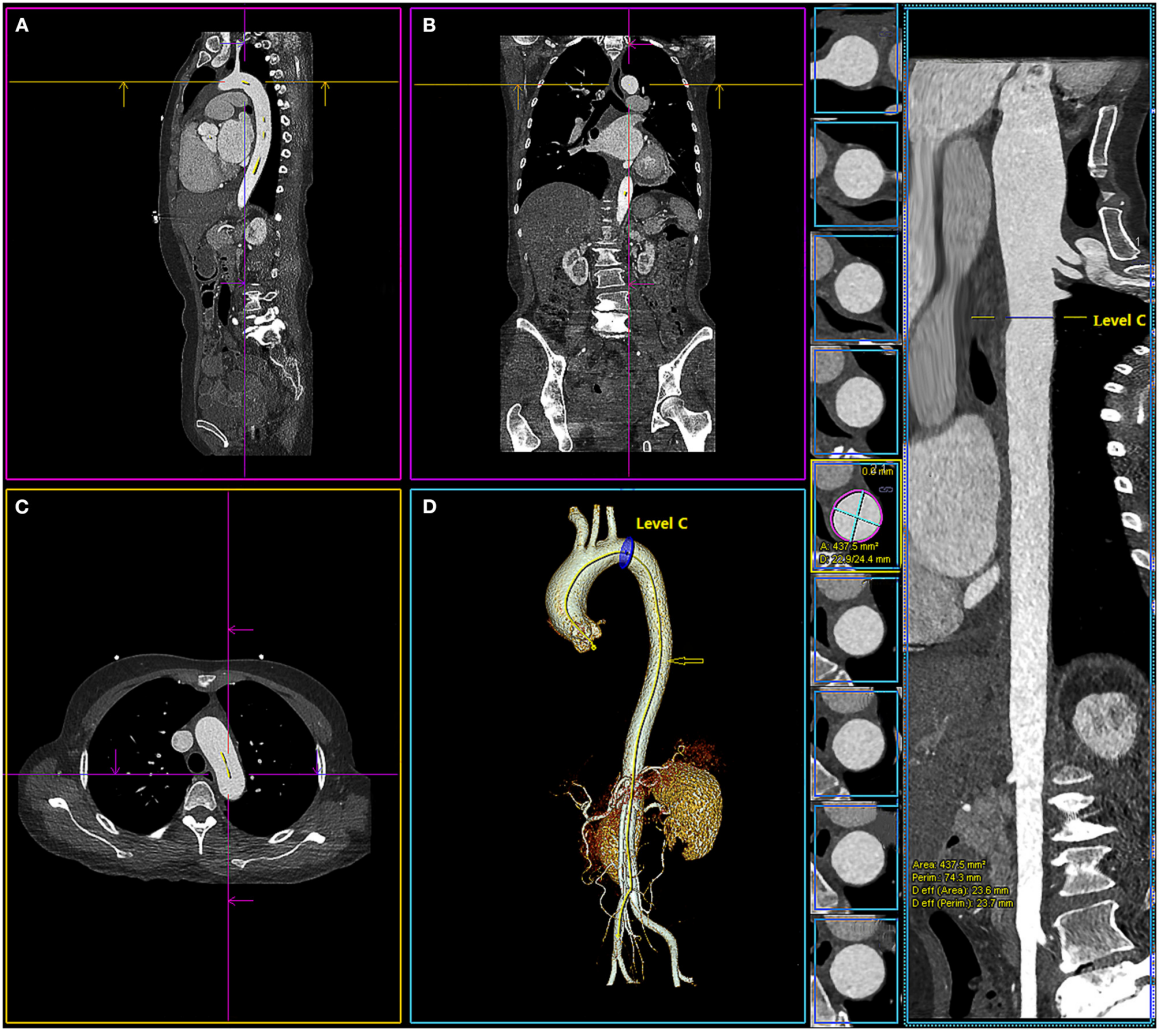
Multiplanar views of aortic measurements in sagittal view **(A)**, frontal **(B)**, axial **(C)**, 3-dimensional **(D)**, and the centerline of the aorta (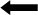) with the location of level C (1 cm distal to the left subclavian artery). The system calculated the maximum and minimum aortic diameter (D: 22.9/24.4 mm), area (area: 437.5 mm^2^), perimeter (perim: 74.3 mm), and diameter derived from the lumen area [D_eff_ (area): 23.6 mm] automatically.

Three segmentations within the aortic arch and proximal descending aorta (level A, 1 cm proximal to the innominate artery; level B, 1 cm distal to the left common carotid artery; and level C, 1 cm distal to the left subclavian artery) were selected, as these were the typical proximal sealing zone for stent-grafts of the thoracic aorta and aortic arch (16) (Figure 2). The morphometric parameter measurements included the maximum aortic diameter, the minimum aortic diameter, the lumen area, the lumen perimeter, and the diameter derived from the lumen area. The measurements were made in a multi-planar view perpendicular to the semi-automatically created centerline. The centerline was adjusted manually in cases where it was not created accurately by the software [when adjusting the centerline manually, the identified centerline should be observed on multi-planar views (axial, coronal, and sagittal) and the cardiopulmonary resuscitation (CPR) image to make sure the centerline was centered in the blood vessel]. If the automatically detected contour of the aorta was incorrect, then it will be manually modified. The system could measure the maximum aortic diameter and minimum aortic diameter on the axial image perpendicular to the centerline and calculated the area, perimeter, and diameter derived from the lumen area automatically (Figure 3; Supplementary Figure S1).

One radiologist with 15-year experience made all the measurements, and another radiologist with 18-year experience who was blinded to the results of the first radiologist made measurements of 20 randomly selected patients at level A. The intraclass correlation coefficient (ICC, two-way random model) and Bland-Altman analysis were calculated to evaluate the inter-observer and intra-observer consistency.

### Statistical Analysis

The categorical variables are expressed as numbers (percentages) and the continuous variables as means ± standard deviation (SD) for all the normally distributed data. All statistical analyses were performed using SPSS 22.0 software (IBM Corporation, Armonk, NY, USA) and GraphPad Prism 8.0 software (GraphPad Software, San Diego, CA, USA), and a value of *p* < 0.05 was considered to be statistically significant. The paired t-test or the Wilcoxon signed-rank test was used to compare the difference of each parameter between systolic and diastolic phases. The mean change in percentage in the maximum aortic diameter, lumen area, perimeter, and diameter derived from lumen area at each level was compared respectively using one-way ANOVA. The ICC was used to evaluate the inter-observer and intra-observer consistency and an ICC >0.80 indicates a high consistency. Moreover, Bland-Altman analysis of bias and limits of agreement were used to describe the agreement of the mean differences.

## Results

**Figure 4:**
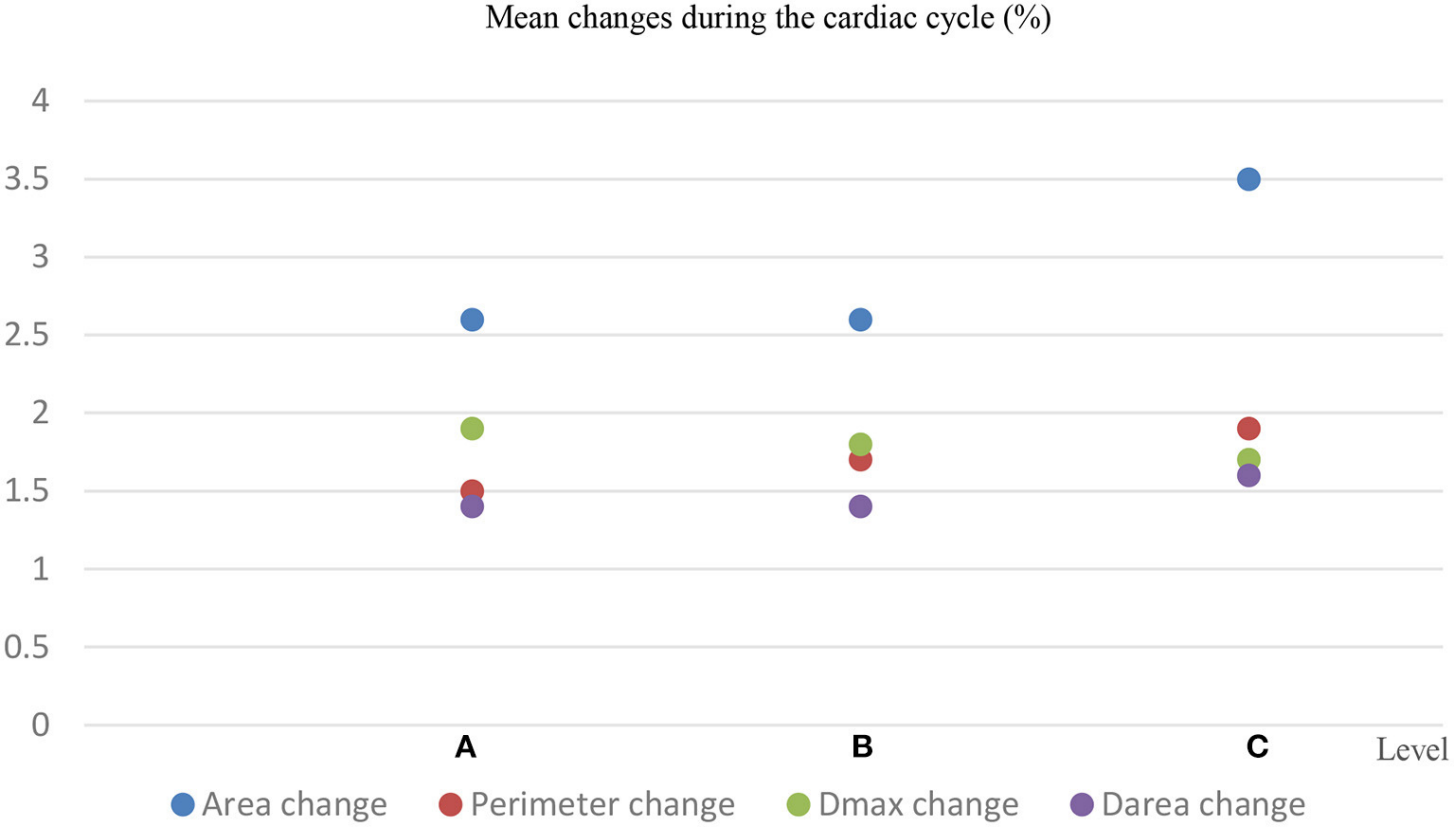
Mean changes in the aortic area, aortic perimeter, maximum diameter (D_max_), and diameter deriving from the lumen area (D_area_) at 3 different locations per cardiac cycle.

The baseline characteristics of these patients are shown in Table 1. There was a significant difference in the aortic dimensions of each measurement parameter between the systolic and diastolic phases (*p* < 0.001; Table 2). The changes per level during the cardiac cycle are shown in Table 3. The mean change in percentage was the smallest in the diameter derived from lumen area at all the three levels when compared to the area, perimeter, and the maximum aortic diameter (1.4 vs. 2.6 vs. 1.5% vs. 1.9 at level A, *p* < 0.01; 1.4 vs. 2.6 vs. 1.7 vs. 1.8% at level B, *p* < 0.01; 1.6 vs. 3.5 vs. 1.9% vs. 1.7 at level C, *p* < 0.01; Figure 4). There was no significant difference in the aortic dimensional change rates of each measurement between the systolic and diastolic phases in patients with or without hypertension (Supplementary Table S1). There was also no significant difference in the aortic dimensional change rates of each measurement when they were grouped by sex and valvular disease (Supplementary Tables S2, S3).

**Table 1:** Baseline characteristics of the patients.

**Variables**	**Patients (*N* = 90)**
Age (years), mean ± SD	60.9 ± 12.4
Male, *n* (%)	68 (75.6)
Female, *n* (%)	22 (24.4)
Blood pressure (mmHg, mean ± SD)	
Systole	129.8 ± 7.7
Diastole	82.3 ± 9.7
Pulse pressure	47.5 ± 7.2
Hypertension, *n* (%)	55 (61.1)
Atherosclerosis, *n* (%)	27 (30.0)
Diabetes mellitus, *n* (%)	16 (17.8)
Smoking, *n* (%)	24 (26.7)
Valvular disease, *n* (%)	55 (61.1)
CAD, *n* (%)	28 (31.1)
AAA/AAD, *n* (%)	12 (13.3)
Atrial fibrillation, *n* (%)	8 (8.9)
Hyperlipidaemia, *n* (%)	4 (4.4)
Hyperuricemia, *n* (%)	5 (5.6)
Hyperthyroidism, *n* (%)	1 (1.1)
Hypothyroidism, *n* (%)	3 (3.3)
COPD, *n* (%)	2 (2.2)

*SD, standard deviation; CAD, coronary arterial disease; AAA, abdominal aortic aneurysm; AAD, abdominal aortic dissection; COPD, chronic obstructive pulmonary disease*.

**Table 2:** The aortic dimensional measurements during the cardiac cycle at different levels.

	**Level**	**D_**max**_ (mm)**	**D_**min**_ (mm)**	**Area (mm^**2**^)**	**Perimeter (mm)**	**D_**area**_ (mm)**
Systolic	A	36.9 ± 4.5	34.2 ± 4.7	999.6 ± 251.5	112.0 ± 14.2	35.4 ± 4.6
	B	31.0 ± 3.8	28.3 ± 3.3	700.0 ± 160.8	94.1 ± 11.5	29.6 ± 3.5
	C	29.0 ± 3.7	26.4 ± 3.3	611.0 ± 150.5	87.8 ± 11.3	27.7 ± 3.5
Diastolic	A	36.2 ± 4.5^*^	33.6 ± 4.6^*^	973.3 ± 244.8^*^	110.3 ± 14.0^*^	34.9 ± 4.5^*^
	B	30.5 ± 3.8^*^	28.0 ± 3.5^*^	682.2 ± 160.0^*^	92.5 ± 11.3^*^	29.2 ± 3.5^*^
	C	28.5 ± 3.7^*^	26.0 ± 3.4^*^	590.3 ± 146.9^*^	86.2 ± 11.1^*^	27.2 ± 3.4^*^

*Data were presented with mean ± standard deviation (SD); D_max_ = the maximum diameter; D_min_ = the minimum diameter; D_area_ = the diameter deriving from the lumen area;*

* *the difference between systole and diastole was statistically significant (p < 0.001)*.

**Table 3:** Changes in mean aortic area, aortic perimeter, maximum diameter, and diameter deriving from the lumen area at three different locations per cardiac cycle.

	**A**	**B**	**C**
Area change (mm^2^)			
Mean (%)	26.3 (2.6)	17.8 (2.6)	20.7 (3.5)
Min (%)	−10.2 (−1.2)	−75 (−7)	−32.8 (−5.3)
Max (%)	85.5 (8.1)	67.8 (7.3)	57.2 (8.4)
SD (%)	19.9 (1.8)	16.4 (2.1)	13.9 (2.2)
Perimeter change (mm)			
Mean (%)	1.7 (1.5)	1.6 (1.7)	1.6 (1.9)
Min (%)	−2.3 (−1.7)	−0.8 (−0.9)	−2.4 (−2.7)
Max (%)	4.4 (3.8)	7.4 (8.4)	6.9 (6.8)
SD (%)	1.3 (1)	1.3 (1.5)	1.2 (1.3)
D_max_ change (mm)			
Mean (%)	0.7 (1.9)	0.5 (1.8)	0.5 (1.7)
Min (%)	−0.8 (−2.3)	−0.4 (−1.3)	−1.3 (−4.3)
Max (%)	6.2 (18.8)	1.7 (6.4)	1.5 (4.9)
SD (%)	0.7 (2.1)	0.3 (1.2)	0.4 (1.4)
D_min_ change (mm)			
Mean (%)	0.5 (1.5)	0.4 (1.4)	0.4 (1.5)
Min (%)	−0.9 (−2.6)	−2.6 (−8.1)	−1.9 (−9.1)
Max (%)	6 (16.2)	3.1 (14.6)	1.5 (7.1)
SD (%)	0.8 (2.3)	0.6 (2.3)	2.1 (1.4)
D_area_ change (mm)			
Mean (%)	0.5 (1.4)	0.4 (1.4)	0.5 (1.6)
Min (%)	−0.3 (−0.8)	−1.1 (−3.2)	−1.7 (−7.8)
Max (%)	1.8 (4.4)	1.7 (7.7)	1.8 (6)
SD (%)	0.4 (1)	1.5 (1.2)	0.4 (1.6)

*SD, standard deviation; D_max_, the maximum diameter; D_min_, the minimum diameter; D_area_, the diameter deriving from the lumen area; Minus value means the dimension in the diastolic phase was larger than that in the systolic phase*.

**Figure 5:**
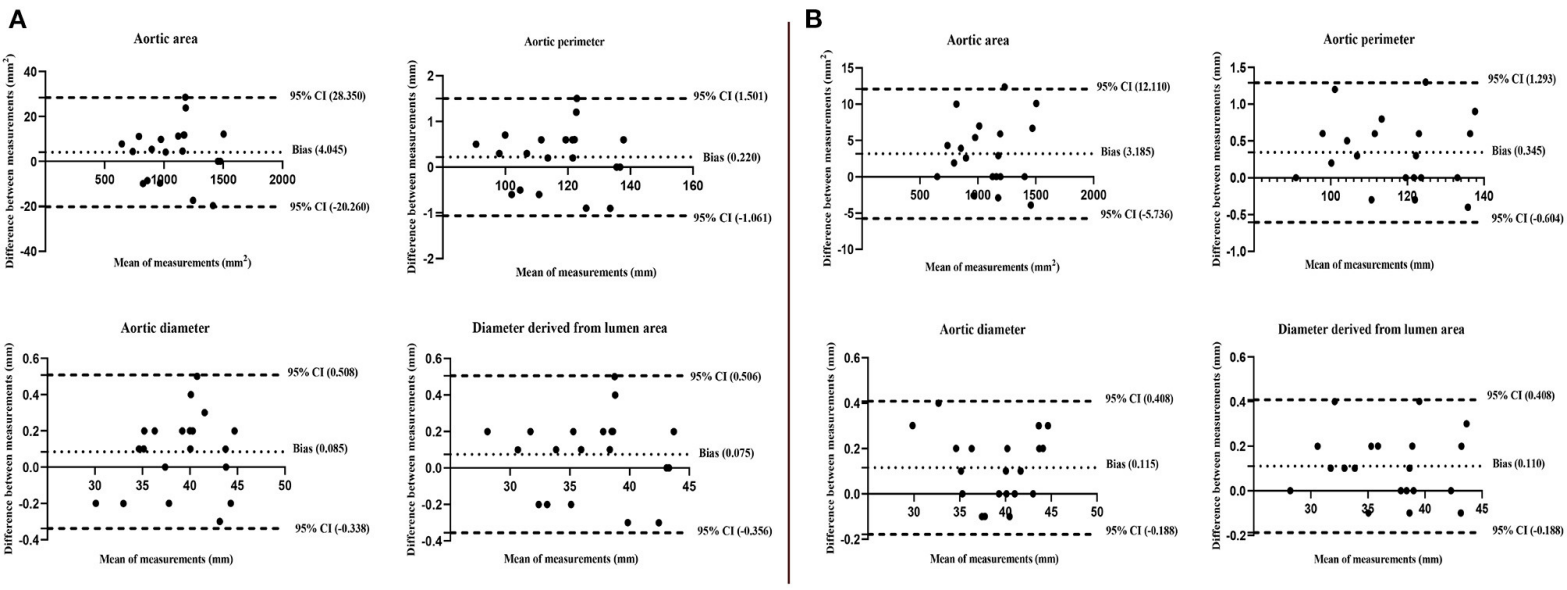
Bland-Altman plots show high consistency of inter-observer **(A)** and intra-observer **(B)** for the aortic area, perimeter, diameter, and diameter derived from the lumen area at level A. The upper and lower limits of each measurement difference during the cardiac cycle were included in the 95% CIs.

The inter-observer analysis showed a high consistency between the two independent observers and the intra-observer analysis also showed a high consistency (Table 4). These results were also confirmed by the Bland-Altman analysis (Figure 5).

**Table 4:** The intraclass correlation coefficient analysis results.

	**Parameter**	**ICC (lower boundary of the CI)**	**MD (mm)**	**SD (mm)**	***p* value**
Inter-observer	Diameter	0.995	0.1	0.2	<0.001
analysis	Area	0.997	4.0	12.0	<0.001
	Perimeter	0.997	0.2	0.6	<0.001
	D_area_	0.997	0.1	0.2	<0.001
Intra-observer	Diameter	0.995	0.1	0.1	<0.001
analysis	Area	0.996	3.2	4.4	<0.001
	Perimeter	0.999	0.3	0.5	<0.001
	D_area_	0.996	0.1	0.1	<0.001

*CI, confidence interval; MD, mean difference; SD, standard deviation*.

## Discussion

Three measurement locations were selected in this study, as all of them were the common proximal landing zones for stent-graft at the aortic arch and proximal descending thoracic aorta in clinical practice. Five different measurement parameters were selected in this study, which included all measurement parameters in current literature, and all these parameters were automatically obtained on the post-processing software with high repeatability. The measurement of each parameter was made based on a centerline method, which has been verified with higher repeatability than unformatted axial images (15). Meanwhile, one purpose of this study was to study whether other measurements rather than the traditional maximum aortic diameter have a smaller change rate between the systolic and diastolic phases and could be used as a reference for stent selection at the aortic arch and proximal descending thoracic aorta, which has been seldom reported, especially at level B (no relevant reports have been published). Thus, our study could verify the previous results at levels A and C and present the regularity of dimensional changes at level B during the cardiac cycle. Overall, the present study had its novelty and relatively high repeatability.

There was a significant difference in the aortic dimensions of each measurement parameter between the systolic and diastolic phases (*p* < 0.001), which confirmed the aortic pulsatility. The reported aortic diameter change during the cardiac cycle in published literature has also yielded controversial results. The aortic diameter change in the ascending aorta was ranged from −3.45 to 27.5%, in the aortic arch ranged from −4.5 to 13.3%, and in the thoracic aorta ranged from −6.02 to 22.6% (10, 11, 16, 17). The possible reasons may result from measurement method differences, measurement location differences, and population selection differences (the number of cases; patients with some factors, which could interfere with the measured results). Moreover, a recent report (18) showed that the inter-observer variability of maximum diameter measurement in the thoracic aorta is 0.13 (−1.92, 2.17) mm, which is quite high as compared to the changes during the cardiac cycle, as reported by this study. Notably, the comparison of the two measures is affected by the variability of both, as stressed in the abovementioned paper. Thus, it cannot be excluded as the relative error in measurements, and this may also be a possible reason. In our study, we included a relatively large cohort and excluded patients with severe calcification of thoracic descending aorta, thoracic aortic disease, previous thoracic aortic surgery, and left ventricular ejection fraction <40%, which could alter the normal hemodynamic of the aorta leading to incorrect measurement (19). Thus, more studies with a relatively larger number of cases and a strict study population selection should be performed to draw convincing results. Moreover, more studies to compare the differences in aortic dimensional measurement between registration-based technique (18) and manual assessment are needed. Of note, 61% of patients in the present study were presented with hypertension, which could have a direct impact on the systolic-diastolic changes. However, there was no significant difference in the aortic dimensional change rates of each measurement between the systolic and diastolic phases in patients with or without hypertension. The reason may be attributed to the different grades of hypertension, anti-hypertension drugs, and the time of hypertension history. Thus, more relevant studies are needed to explore the exact effect of hypertension on aortic dimensional change during the systolic and diastolic phases.

Belvroy et al. (10) reported that the changes were the smallest in the aortic area when compared to the diameter and perimeter at the ascending aorta, and thus the authors recommended measuring the area when selecting the stent-graft size at this location. Parodi et al. (11) found that there was a significant difference between measuring the diameter directly and deriving it from the lumen area (*p* < 0.001) and advocated that the aortic diameter derived from the lumen area should be selected as a reference when choosing desired oversizing for stent-graft in the descending aorta. Thus, other parameters, such as the area in comparison to diameter, may potentially provide a more accurate size reference for stent selection. In the present study, the diameter derived from the lumen area at all three levels was changed least over time when compared to the area, perimeter, and the maximum aortic diameter (1.4 vs. 2.6 vs. 1.5 vs. 1.9% at level A, *p* < 0.01; 1.4 vs. 2.6 vs. 1.7 vs. 1.8% at level B, *p* < 0.01; 1.6 vs. 3.5 vs. 1.9 vs. 1.7% at level C, *p* < 0.01). Therefore, the aortic diameter derived from the lumen area over other measurement parameters may provide a better evaluation for selecting the size of the stent at the aortic arch and the proximal thoracic descending aorta. However, a prospective study comparing these different measurement parameters regarding the clinical outcomes was still needed to evaluate the clinical implications. In addition, the maximum change of diameter derived from the lumen area ranges from 1.7 to 7.7%, thus 10% oversizing might be more reasonably preferred rather than 20%, which is consistent with Guo's study (16).

Our study had some limitations. First, the comparison of the dimension was only performed in partial segments of the aorta. Thus, we plan to analyze other segments in further study. Second, the sample number in this series was small and a larger scale study was needed to make the results more representative. Third, the results need to be verified in clinical practice.

## Conclusion

The present study demonstrated that the aortic dimensional differences during the cardiac cycle are significant and the aortic diameter derived from the lumen area over other measurement parameters may provide a better evaluation for selecting the size of the stent at the aortic arch and the proximal thoracic descending aorta. In addition, a prospective study comparing these different measurement parameters regarding the outcomes is still needed to evaluate the clinical implications.

## Data Availability Statement

The original contributions presented in the study are included in the article/Supplementary Material, further inquiries can be directed to the corresponding author/s.

## Ethics Statement

The studies involving human participants were reviewed and approved by the Institutional Ethics Review Board of Wuhan Union Hospital. Written informed consent for participation was not required for this study in accordance with the national legislation and the institutional requirements.

## Author Contributions

BX, WZ, YW, and YC contributed to the design, methodology, investigation, and draft writing. JL, QS, SH, and CY contributed to data collection, data analysis, and draft review. TL and CZ contributed to data collection, data analysis, and editing. BX contributed to conception and design, methodology, data interpretation, and draft revising. All authors contributed to the article and approved the submitted version.

## Funding

This work was funded by Grants from the National Natural Science Foundation of China (81873917) and the China Health Promotion Foundation (XM_2018_011_0006_01).

## Conflict of Interest

The authors declare that the research was conducted in the absence of any commercial or financial relationships that could be construed as a potential conflict of interest.

## Publisher's Note

All claims expressed in this article are solely those of the authors and do not necessarily represent those of their affiliated organizations, or those of the publisher, the editors and the reviewers. Any product that may be evaluated in this article, or claim that may be made by its manufacturer, is not guaranteed or endorsed by the publisher.
