# Supplementary material for: Dynamic Changes in the Aorta During the Cardiac Cycle Analyzed by ECG-Gated Computed Tomography
Source: Front Cardiovasc Med. 2022 May 19;9:793722. doi: 10.3389/fcvm.2022.793722 (PMC9160308; doi:10.3389/fcvm.2022.793722)
Supplement: Supplementary file 1 [file Data_Sheet_1.docx]

Supplementary Figure 1.


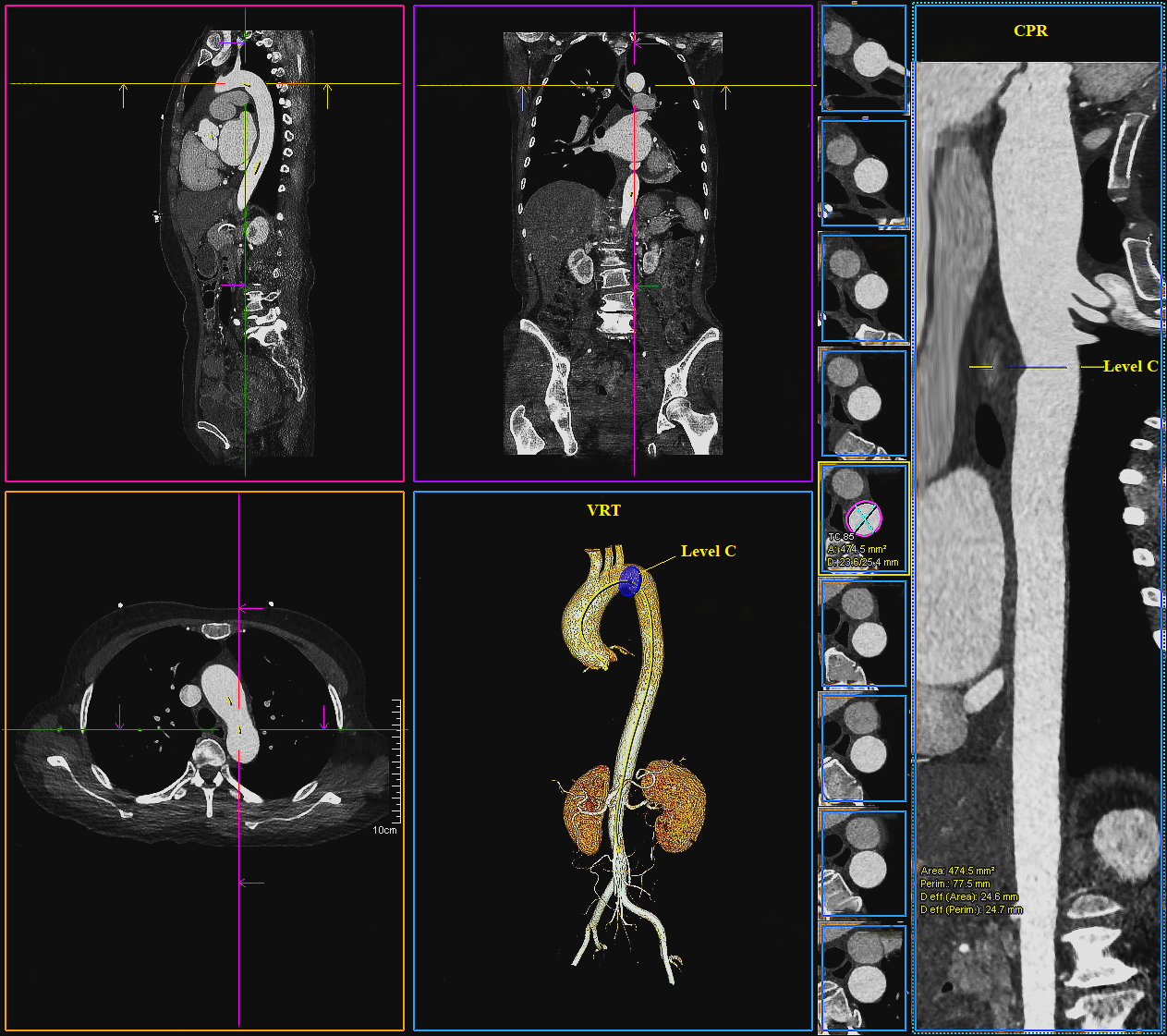


Legends: The measurement levels were selected on the curved planar reformation (CPR) image which could visualize a curved planar reconstruction of a vessel along a defined centerline. In the toolbar of the CPR segment, click the Vessel Length Measurement icon. Then, a green and a red marker appear which represent the upper and lower border of the measurement. These markers are visualized by squared discs in the VRT (volume rendering technique). The measured section is highlighted by a blue line in the VRT and the CPR segments. The measured length between both reference markers is displayed in the CPR segment. Thus, we can measure the curved distances along the vessel path, for example, if you want to determine the distance from the ostium of the vessel to a predefined level. Similarly, we can accurately identify a predefined measurement level, for example, 1 cm distance distal to the left subclavian artery (Level C). Then the system could measure the maximum and minimum aortic diameter on axial image perpendicular to the centerline, and calculated the area, perimeter, and diameter derived from the lumen area automatically.

Supplementary Table 1. The change rate of each measurement between systolic and diastolic phases in patients with or without hypertension.

| Level | Variables | Change rate (%),mean ± SD | | *P* value |
| --- | --- | --- | --- | --- |
|  |  | Hypertension | No hypertension |  |
| A | Area | 2.4±1.7 | 3.0±1.8 | 0.158 |
|  | Perimeter | 1.4±1.1 | 1.6±0.9 | 0.393 |
|  | Dmax | 1.9±2.6 | 1.9±1.1 | 0.934 |
|  | Dmin | 1.3±1.7 | 1.9±3.0 | 0.262 |
|  | Darea | 1.3±1.0 | 1.5±0.9 | 0.557 |
| B | Area | 2.4±2.2 | 3.0±1.9 | 0.214 |
|  | Perimeter | 1.7±1.5 | 1.7±1.4 | 0.812 |
|  | Dmax | 1.7±1.1 | 1.9±1.3 | 0.124 |
|  | Dmin | 1.1±1.9 | 1.9±2.8 | 0.372 |
|  | Darea | 1.2±1.1 | 1.7±1.4 | 0.075 |
| C | Area | 3.2±2.4 | 3.9±2.0 | 0.151 |
|  | Perimeter | 1.7±1.4 | 2.2±1.1 | 0.058 |
|  | Dmax | 1.7±1.4 | 1.7±1.5 | 0.907 |
|  | Dmin | 1.4±1.9 | 1.6±2.5 | 0.759 |
|  | Darea | 3.2±2.4 | 3.9±2.0 | 0.466 |

SD= standard deviation; Dmax=the maximum diameter; Dmin=the minimum diameter; Darea= the diameter deriving from the lumen area; Change rate= (systolic phase value-diastolic phase value)/ systolic phase value ×100%.

Supplementary Table 2. The change rate of each measurement between systolic and diastolic phases in male patients or female patients.

| Level | Variables | Change rate (%) | | *P* value |
| --- | --- | --- | --- | --- |
|  |  | Male | Female |  |
| A | Area | 2.4±1.6 | 3.2±2.1 | 0.073 |
|  | Perimeter | 1.4±1.1 | 1.6±1.0 | 0.368 |
|  | Dmax | 2.0±2.3 | 1.8±1.2 | 0.776 |
|  | Dmin | 1.5±2.5 | 1.6±1.5 | 0.830 |
|  | Darea | 1.3±0.9 | 1.5±1.0 | 0.393 |
| B | Area | 2.6±1.6 | 2.8±3.2 | 0.682 |
|  | Perimeter | 1.6±1.3 | 2.0±1.9 | 0.205 |
|  | Dmax | 1.7±1.2 | 1.8±1.1 | 0.740 |
|  | Dmin | 1.3±2.4 | 1.6±2.2 | 0.703 |
|  | Darea | 1.3±1.2 | 1.8±1.2 | 0.126 |
| C | Area | 3.4±2.3 | 3.7±2.1 | 0.645 |
|  | Perimeter | 1.8±1.4 | 2.0±1.0 | 0.484 |
|  | Dmax | 1.7±1.2 | 1.5±2.0 | 0.653 |
|  | Dmin | 1.6±1.9 | 1.3±2.8 | 0.584 |
|  | Darea | 1.7±1.2 | 1.5±2.4 | 0.670 |

SD= standard deviation; Dmax=the maximum diameter; Dmin=the minimum diameter; Darea= the diameter deriving from the lumen area; Change rate= (systolic phase value-diastolic phase value)/ systolic phase value ×100%.

Supplementary Table 3. The change rate of each measurement between systolic and diastolic phases in patients with or without valvular disease.

| Level | Variables | Change rate (%) | | *P* value |
| --- | --- | --- | --- | --- |
|  |  | Valvular disease | No Valvular disease |  |
| A | Area | 2.6±1.8 | 2.6±1.8 | 0.987 |
|  | Perimeter | 1.6±1.1 | 1.4±1.1 | 0.439 |
|  | Dmax | 2.3±3.0 | 1.7±1.2 | 0.154 |
|  | Dmin | 1.6±3.1 | 1.5±1.6 | 0.763 |
|  | Darea | 1.3±0.9 | 1.4±1.0 | 0.645 |
| B | Area | 2.7±2.3 | 2.6±1.9 | 0.922 |
|  | Perimeter | 2.0±1.9 | 1.5±1.1 | 0.070 |
|  | Dmax | 1.8±1.5 | 1.7±1.0 | 0.691 |
|  | Dmin | 1.7±2.6 | 1.2±2.2 | 0.381 |
|  | Darea | 1.6±1.3 | 1.3±1.2 | 0.328 |
| C | Area | 3.6±2.7 | 3.4±1.9 | 0.618 |
|  | Perimeter | 1.9±1.6 | 1.8±1.1 | 0.724 |
|  | Dmax | 1.8±1.4 | 1.6±1.4 | 0.510 |
|  | Dmin | 1.9±2.2 | 1.3±2.0 | 0.187 |
|  | Darea | 1.7±1.3 | 1.6±1.7 | 0.781 |

SD= standard deviation; Dmax=the maximum diameter; Dmin=the minimum diameter; Darea= the diameter deriving from the lumen area; Change rate= (systolic phase value-diastolic phase value)/ systolic phase value ×100%.
